# Supplementary material for: Dental microwear reveals mammal-like chewing in the neoceratopsian dinosaur Leptoceratops gracilis
Source: PeerJ. 2016 Jul 6;4:e2132. doi: 10.7717/peerj.2132 (PMC4941762; doi:10.7717/peerj.2132)
Supplement: Article S1 [file peerj-04-2132-s001.docx]

**Supplemental Information**

**Macro instructions and code**

Microsoft Excel Macro for transforming Cartesian coordinate files generated by Microware 4.02 into angles, lengths and widths.

**Instructions for using the macro in Microsoft Excel 2013**

1. Download the Excel file with attached macro, or create a macro in Excel by copying the below code into the macro editor.
2. If prompted by Excel, allow “Enable editing” and “Content” or the macro will not function.
3. Click the “DATA” tab, next click on “From Text” in the “Get External Data” menu.
4. Change to chooser in the lower right corner from “Text Files” to “All Files” otherwise the Cartesian coordinate files will not be available for selection. Go to the desired folder containing your coordinate files and click on one. Once it is highlighted in the “File name:” choose click import.
5. The “Text Import Wizard” will pop-up with “Step 1 of 3” and the radio button “Delimited” should be preselected on. If it is not, then select it and click “Next”
6. On “Step 2” the “Tab” radio button should be preselected. If it is not, select it as well as the “Comma” radio button, and then click “Next”
7. On “Step 3” the “General” radio button should be preselected, if it is not select it and click “Next”.
8. A small “Import Data” window will appear. Under “Where do you want to put the data?” confirm that the “Existing worksheet” radio button is selected and in the space provided give the cell registration as =$A$1. If the first cell of the first column is not listed here as the registration then the macro will not function.
9. Click “OK” and your data should import into Excel.
10. Now go to the “VIEW” tab and in the far right click the “Macros” icon. A window with the macro will appear. Select the named macro by clicking on it and then click “Run”. You should see the macro execute its code by performing calculations. The output will spool and the resulting data will appear in columns. See the below description for details and interpretation of the column contents.

**Description of column contents in macro output, and how to alter code in specific subroutines.**

**Column A:** Is the feature number as recorded in the coordinate file sequentially during digitization with Microware 4.02.

**Columns B-I:** X1, Y1 and X2, Y2 and their associated primes are the Cartesian coordinates from the coordinate file produced by Microware 4.02.

**Columns J, and K:** Labeled “Linear1” and “Linear 2” are the linear dimensions of features in pixels. Linear 1 is the linear dimension of the line described by coordinates in columns B-E (X1, Y1; X1’, Y1’). Linear 2 is the dimension of the coordinates in columns F-I (X2, Y2; X2, Y2).

**Columns L, and M**: Labelled “Length” and “Breadth” are the maximum and minimum linear dimensions from columns J and K now sorted into their own column. This determines which of the two measurements of a feature is the longest, and consequently allows determination of which is the length and the breadth.

**Column N**: is the ratio of Length to Breadth. This column is used to define a feature as a pit or a scratch. Any feature with a length to breadth ratio > or = 4 is considered a scratch and its angle of intersection with the apico-basal axis will be calculated. If it is less than the cutoff value it is considered a pit and its angle is not calculated.

**Columns O –T**: X3, Y3 and associated primes are the Cartesian coordinates used to define the corners of the right triangle that will be used to derive angles. You will notice that the first two sets of coordinates, Columns O-R (X3, Y3 and X3’, Y3’) are the coordinates of the length dimension that is sorted from columns B-I. These coordinates define the hypotenuse. Columns S and T (X3’’, Y3’’) are the coordinates of the corner of the right triangle. They are derived from Columns Q and P, labelled X3’and Y3 respectively. They will always be the lesser Y coordinates, and so this point of the triangle will always be closest to the X axis

**Columns U-W**: Enumerate the length of the legs of the right triangle labelled B and C and the length of the hypotenuse (A (Hyp)) calculated from the Cartesian coordinates in columns O-T. The hypotenuse is calculated using the Pythagorean Theorem on the lengths and it should always be equal with the value in column L (Length). This comparison certifies that the macro has computed values correctly up to this point.

**Columns X, and Y:** Labelled “Radians” and “Degrees” are the angle of the right triangle measured in radians and degrees thought a 180° arch from left to right through the bottom hemisphere of a micrograph.

**Columns Z-AC:** Labelled “Angle”, “EuLenght”, “Euwidth”, and “Feature #” these columns list the desired data this macro was created to extract. These columns are sorted so that any blank cells in the “Degrees” column that would be associated with pits fall to the bottom of the columns. This sorts the angle value of a feature (Feature #) and its associated length and width to the top of the column in decreasing angle values. “EuLenght” and “Euwidth” are the true length and width of features measured in micrometers and calculated by converting the pixel lengths from columns L (Length) and M (Breadth) using a conversion factor. This conversion factor is dependent on the magnification and dpi of a micrograph, and must be changed within the macro code to yield accurate values in columns AA and AB if these properties are different from those the macro was written for. To adjust the macro code, find lines 227 and 238 in the titled subroutines “Finding the true length (width) of a feature” change the value 0.846667 to whatever value is the conversion factor for your image based on the following equation. Conversion factor = 25,400 µm per inch / (Magnification x dpi). For instance, the value currently listed is 0.846667µm/pixel, but an image with properties of 500x and 200dpi would have a value of 0.254µm/pixel = 25,400/(500 x 200).

**Macro Code**

Sub Angle300Dpi100x()

' Microwear orientation angles

' Macro recorded 2/15/2006-2016

' By Frank J. Varriale

' King's College

' 113 North River Street

' Wilkes-Barre, Pennsylvania 18711, USA

' 1(570)208-5900

' [frankvarriale@kings.edu](mailto:frankvarriale@kings.edu)

'Card File Cartesian Cordiante Lables

Range("A1:AC1").Select

Selection.Font.Bold = True

Range("a1") = "Feature #"

Range("B1") = "X1"

Range("C1") = "Y1"

Range("D1") = "X1'"

Range("E1") = "Y1'"

Range("F1") = "X2"

Range("G1") = "Y2"

Range("H1") = "X2'"

Range("I1") = "Y2'"

'Linear1

Range("J1") = "Linear1"

Range("J2").Select

Do Until Selection.Offset(0, -9).Value = ""

Selection.Value = "=SQRT((RC[-8]-RC[-6])^2+(RC[-7]-RC[-5])^2)"

Selection.Offset(1, 0).Select

Loop

'Linear2

Range("K1") = "Linear2"

Range("K2").Select

Do Until Selection.Offset(0, -10).Value = ""

Selection.Value = "=SQRT((RC[-5]-RC[-3])^2+(RC[-4]-RC[-2])^2)"

Selection.Offset(1, 0).Select

Loop

'MaxLinear & MinLinear

Range("L1") = "Length"

Range("M1") = "Breadth"

Range("J2").Select

Do Until Selection.Offset(0, -9).Value = ""

If Selection.Value > Selection.Offset(0, 1) Then

Selection.Offset(0, 2).Value = Selection.Value

Selection.Offset(0, 3).Value = Selection.Offset(0, 1)

Else

Selection.Offset(0, 2).Value = Selection.Offset(0, 1)

Selection.Offset(0, 3).Value = Selection.Value

End If

Selection.Offset(1, 0).Select

Loop

'Ratio

Range("N1") = "Ratio"

Range("N1").Select

Selection.Font.ColorIndex = 3

Range("N2").Select

Do Until Selection.Offset(0, -13).Value = ""

ActiveCell.FormulaR1C1 = "=RC[-2]/RC[-1]"

Selection.Offset(1, 0).Select

Loop

'Find Striations & Reorder Their Cartesian Cordinates

Range("O1") = "X3"

Range("P1") = "Y3"

Range("Q1") = "X3'"

Range("R1") = "Y3'"

Range("N2").Select

Do Until Selection.Offset(0, -13).Value = ""

If Selection.Value >= 4 And Selection.Offset(0, -4).Value > Selection.Offset(0, -3).Value Then

If Selection.Offset(0, -11).Value < Selection.Offset(0, -9).Value Then

Selection.Offset(0, 1).Value = Selection.Offset(0, -12).Value

Selection.Offset(0, 2).Value = Selection.Offset(0, -11).Value

Selection.Offset(0, 3).Value = Selection.Offset(0, -10).Value

Selection.Offset(0, 4).Value = Selection.Offset(0, -9).Value

Else

Selection.Offset(0, 1).Value = Selection.Offset(0, -10).Value

Selection.Offset(0, 2).Value = Selection.Offset(0, -9).Value

Selection.Offset(0, 3).Value = Selection.Offset(0, -12).Value

Selection.Offset(0, 4).Value = Selection.Offset(0, -11).Value

End If

End If

Selection.Offset(1, 0).Select

Loop

Range("N2").Select

Do Until Selection.Offset(0, -13).Value = ""

If Selection.Value >= 4 And Selection.Offset(0, -4).Value < Selection.Offset(0, -3).Value Then

If Selection.Offset(0, -7).Value < Selection.Offset(0, -5).Value Then

Selection.Offset(0, 1).Value = Selection.Offset(0, -8).Value

Selection.Offset(0, 2).Value = Selection.Offset(0, -7).Value

Selection.Offset(0, 3).Value = Selection.Offset(0, -6).Value

Selection.Offset(0, 4).Value = Selection.Offset(0, -5).Value

Else

Selection.Offset(0, 1).Value = Selection.Offset(0, -6).Value

Selection.Offset(0, 2).Value = Selection.Offset(0, -5).Value

Selection.Offset(0, 3).Value = Selection.Offset(0, -8).Value

Selection.Offset(0, 4).Value = Selection.Offset(0, -7).Value

End If

End If

Selection.Offset(1, 0).Select

Loop

'Create Trinagle Corner Cartesian Cordinates

Range("S1") = "X3''"

Range("S2").Select

Do Until Selection.Offset(0, -18).Value = ""

Selection.Value = Selection.Offset(0, -2).Value

Selection.Offset(1, 0).Select

Loop

Range("T1") = "Y3''"

Range("T2").Select

Do Until Selection.Offset(0, -19).Value = ""

Selection.Value = Selection.Offset(0, -4).Value

Selection.Offset(1, 0).Select

Loop

'Calcualate Triangle Leg Lengths

'B leg

Range("U1") = "B"

Range("U2").Select

Do Until Selection.Offset(0, -20).Value = ""

If Selection.Offset(0, -1) = "" Then

Else

Selection.Value = Abs(Selection.Offset(0, -3).Value - Selection.Offset(0, -1))

End If

Selection.Offset(1, 0).Select

Loop

'C

Range("V1") = "C"

Range("V2").Select

Do Until Selection.Offset(0, -21).Value = ""

If Selection.Offset(0, -2) = "" Then

Else

Selection.Value = Abs(Selection.Offset(0, -7).Value - Selection.Offset(0, -3))

End If

Selection.Offset(1, 0).Select

Loop

'A (Hypotenuse)

Range("W1") = "A (Hyp)"

Range("W2").Select

Do Until Selection.Offset(0, -22).Value = ""

If Selection.Offset(0, -3) = "" Then

Else

Selection.Value = "=SQRT((RC[-2]^2)+(RC[-1]^2))"

End If

Selection.Offset(1, 0).Select

Loop

'Calculate Radians

Range("X1") = "Radians"

Range("X2").Select

Do Until Selection.Offset(0, -23).Value = ""

If Selection.Offset(0, -9) = "" Then

ElseIf Selection.Offset(0, -8).Value = Selection.Offset(0, -6).Value Then

ActiveCell.FormulaR1C1 = "=RADIANS(0)"

ElseIf Selection.Offset(0, -9).Value = Selection.Offset(0, -7).Value Then

ActiveCell.FormulaR1C1 = "=RADIANS(90)"

ElseIf Selection.Offset(0, -9).Value > Selection.Offset(0, -7).Value Then

ActiveCell.FormulaR1C1 = "=ASIN(RC[-3]/RC[-1])"

ElseIf Selection.Offset(0, -9).Value < Selection.Offset(0, -7).Value Then

ActiveCell.FormulaR1C1 = "=RADIANS(180)-ASIN(RC[-3]/RC[-1])"

End If

Selection.Offset(1, 0).Select

Loop

'Calculate Angles

Range("Y1") = "Degrees"

Range("Y1").Select

Selection.Font.ColorIndex = 3

Range("Y2").Select

Do Until Selection.Offset(0, -24).Value = ""

If Selection.Offset(0, -10) = "" Then

ElseIf Selection.Offset(0, -9).Value = Selection.Offset(0, -7).Value Then

ActiveCell.FormulaR1C1 = 0

ElseIf Selection.Offset(0, -10).Value = Selection.Offset(0, -8).Value Then

ActiveCell.FormulaR1C1 = 90

ElseIf Selection.Offset(0, -10).Value > Selection.Offset(0, -8).Value Then

ActiveCell.FormulaR1C1 = "=ROUND(DEGREES(RC[-1]),1)"

ElseIf Selection.Offset(0, -10).Value < Selection.Offset(0, -8).Value Then

ActiveCell.FormulaR1C1 = "=ROUND(DEGREES(RC[-1]),1)"

End If

Selection.Offset(1, 0).Select

Loop

'Creates a colum of Angles next to Degrees and a feature numbers list in last column

Range("AC1") = "Feature #"

Range("Z1") = "Angle"

Range("Z1").Select

Selection.Font.ColorIndex = 3

Range("Z2").Select

Do Until Selection.Offset(0, -25).Value = ""

Selection.Value = Selection.Offset(0, -1).Value

Selection.Offset(0, 3).Value = Selection.Offset(0, -25).Value

Selection.Offset(1, 0).Select

Loop

'Finding the true length of a feature

Range("AA1") = "EuLength"

Range("AA1").Select

Selection.Font.ColorIndex = 16

Range("L2").Select

Do Until Selection.Value = ""

Selection.Offset(0, 15).Value = Round(Selection.Value * 0.846667, 1)

Selection.Offset(1, 0).Select

Loop

'Finding the true width of a feature

Range("AB1") = "EuWidth"

Range("AB1").Select

Selection.Font.ColorIndex = 17

Range("M2").Select

Do Until Selection.Value = ""

Selection.Offset(0, 15).Value = Round(Selection.Value * 0.846667, 1)

Selection.Offset(1, 0).Select

Loop

'Sorts Angles. Length and Width and Removes Spaces

Range("Z2:AC700").Select

Selection.Sort Key1:=Range("Z2"), Order1:=xlDescending, Header:=xlGuess, _

OrderCustom:=1, MatchCase:=False, Orientation:=xlTopToBottom, _

DataOption1:=xlSortNormal

'Centering of values within the cells

Cells.Select

With Selection

.HorizontalAlignment = xlCenter

.VerticalAlignment = xlBottom

.WrapText = False

.Orientation = 0

.AddIndent = False

.IndentLevel = 0

.ShrinkToFit = False

.ReadingOrder = xlContext

.MergeCells = False

End With

Cells.Select

Selection.Columns.AutoFit

Cells.Select

Selection.Rows.AutoFit

Range("AA1").Select

End Sub
